# The Relevance of the Use of Radiographic Planning in Order to Avoid Complications in Mandibular Implantology: A Retrospective Study

**DOI:** 10.1155/2016/8175284

**Published:** 2016-05-12

**Authors:** Gilberto Sammartino, Juan Carlos Prados-Frutos, Francesco Riccitiello, Pietro Felice, Vincenzo Cerone, Roberta Gasparro, Hom-Lay Wang

**Affiliations:** ^1^Department of Neuroscience, Reproductive Sciences and Odontostomatology, University of Naples, Via Pansini 5, Edificio 14, 80131 Naples, Italy; ^2^Stomatology Department, Rey Juan Carlos University, Madrid, Spain; ^3^Department of Periodontology and Implantology, University of Bologna, Bologna, Italy; ^4^School of Dentistry, University of Michigan, Ann Arbor, MI, USA

## Abstract

The aim of this retrospective radiological study was to evaluate the variability of the mandibular anatomy in the presence and absence of teeth and to consider how it could influence implant planning. 187 mandibular CT DentaScans were selected from our department archive according to the inclusion criteria. The axial height, vertical height, angulation of the bone crest, and the bone available for ideal implant placement were measured. The analysis of the data shows that the mandible contour presents a constant degree of angulation. The variation of angulation in the absence of teeth was statistically significant only in the region between the canine and the first premolar and in that between the second premolar and the first molar. The difference between the crest height and the available distance to place the implant was greater in the region of the second molar while in the other regions the implant planning was made complex by postextraction resorption. Alveolar bone resorption after tooth loss can be considered as a risk factor for lingual cortical perforation during the insertion of an implant. To avoid potential intra/postoperative complications, 3D radiographic examination is recommended in order to study the mandibular anatomy and identify the risk areas.

## 1. Introduction

For a better implant placement and to avoid surgical complications, clinicians must have total knowledge of the bone anatomy, including the osseous topography, bone volume, crest angulation, and bone deficiencies [1, 2]. To obtain this information, as well as the clinical examination, a radiographic study of the jaw anatomy is essential. A variety of imaging modalities is available for preoperative planning purposes [3]. Computed Tomography (CT), in the past, and Cone Beam Computed Tomography (CBCT), nowadays, seem to be the best presurgical radiographic evaluations for the prevention of complications related to an incorrect implant placement, either mechanical, esthetic, or surgical. Mechanical complications are caused by the necessity of using angled abutments to correct the implant axis, which results in a bending movement of the implant and potential biomechanical problems [4]. Types of failure relating to angled abutments include fractures of the coating material [5], fractures in parts of the framework [5], loosening of the abutment screws [4, 5], and loss of implant osseointegration [5, 6]. Surgical complications may occur during or immediately after the surgery. They mainly consist in damage to the neurovascular structures close to the implant site [7]. Generally, dental implant positioning in the interforaminal region of an edentulous mandibular ridge is considered a safe surgical procedure [8–10]. The arterial blood supply to the mouth floor is formed by an anastomosis between the sublingual artery (2 mm in average diameter), a branch of the lingual artery, and the submental artery (2 mm in average diameter), a branch of the facial artery [11, 12], which passes close to the lingual plate. Intraosseous hemorrhage is not a serious event and its control can be ensured by compressing the area with an implant, for example [12].

Severe bleeding and the formation of massive hematomas in the floor of the mouth are the result of an arterial trauma. A hemorrhage noted in the mandibular floor of the mouth during or after implant surgery is caused, in most cases, by a perforation of the lingual cortical plate [13]. Goodacre et al. found hemorrhage as a complication of implant surgery placement with an incidence of up to 24% [14]. This occurrence may lead to extensive bleeding into the submandibular area, resulting in a life-threatening acute airway obstruction within the first few hours after surgery [15]. The hemorrhage can easily spread into the loose tissues of the floor of the mouth, the sublingual area, and the space between the lingual muscles, which may require intubation or an emergency tracheostomy [14].

In the posterior mandibular region, major complications are related to damage to the inferior alveolar nerve as well as the mylohyoid artery, a branch of the inferior alveolar artery. The lingual concavity (submandibular gland fossa and submandibular fossa) below the mylohyoid ridge, with its variations, could restrict the implant placement [16].

The alveolar bone crest is subject to vertical and horizontal resorption secondary to tooth loss; this may cause an increased incidence of complications during implant surgery. To avoid these potential complications, an anatomical study of the mandible and its variability is necessary. To perform a 3-dimensional (3D) study of the mandibular bone morphology and to obtain a millimeter (mm) scale read on the vertical and horizontal measurements, CT dental scan examination has become popular [17]. Hence, the aim of this retrospective radiological study was to evaluate the variability of the mandibular anatomy in the presence and absence of teeth and to consider how it could influence implant planning.

## 2. Materials and Methods

### 2.1. CT Selection

Two hundred and twenty-six (out of 513) images {124 males and 102 females; mean age: 45.24 ± 12.86 (range: 18–70)} from the radiographic archive of the Department of Oral Surgery of the University of Naples Federico II that met the following inclusion criteria were obtained:

- A good quality of the CT images (clear, well defined, without any artifacts and interference due to previous treatment such as dental implants, crowns, or pontics).
- An analogical CT presenting a reference plane parallel to the occlusal plane in mandibles with teeth or parallel to the mandibular lower margin in edentulous mandibles and/or a digital CT which provides an original DICOM file (gantry tilt = 0°).
- The presence of teeth in the dental arch or the absence of teeth in the dental arch with a minimum residual height of 10 mm from the alveolar canal and a minimum ridge width of 6 mm, excluding severe atrophies.
- An orthoradial section of 2 mm.
- The absence of pathological or traumatic conditions that might modify the anatomical mandibular morphology.

As the CTs came from different radiological centers, it was hypothesized that the parameters of the patient positioning, exposure, and reconstruction algorithms would be different. Therefore, the CTs were grouped according to the radiological centers, excluding those centers that did not provide a sufficient number of CT scans for the statistical analysis, resulting in a total number of 187 CTs (92 males and 95 females; mean age: 46.78 ± 14.36; age range: 18–70). Finally, given that the measurements for each center were similar to each other, they were grouped into a single sample.

Informed consent was obtained from all patients.

This study has been approved by the Ethical Committee of the University of Naples Federico II.

### 2.2. Procedure

**Figure 1:**
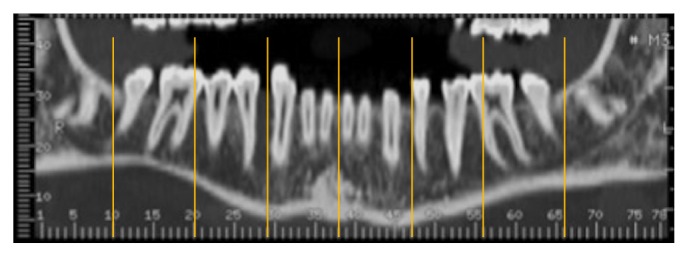
Regions of interest.

The analogical CT images were digitalized using an Epson Perfection 4990 scanner (Seiko Epson Corporation,* Suwa*,* Nagano*, Japan). All the digital CTs were postprocessed to obtain a gantry tilt = 0°. All the measurements were performed on orthoradial sections by a single implantologist (RG) using the digital program OsiriX 5.8.2 for Mac OS X that is recommended for preoperative planning [18]. For each sample, in the presence of teeth, it was decided to perform the measurements in different regions (Figure 1):

- The symphysis region that corresponds to the section between the two central incisors.
- The region between the canine and first premolar on the right and left.
- The region between the second premolar and the first molar on the right and left.
- The region on the distal margin or distal root of the second molar on the right and left.

**Figure 2:**
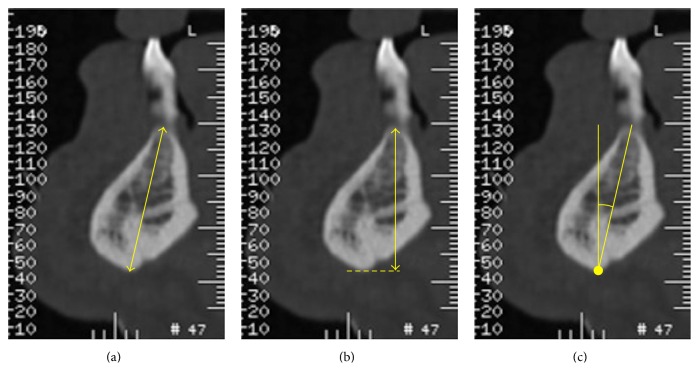
(a) The distance between the most coronal and medial point of the ridge and the lowest point along the axis of the crest; (b) the height from the most coronal and medial point of the ridge to the projection of the lowest point on the perpendicular to the CT reference plane; (c) the angle between the axis of the crest and the line perpendicular to the CT reference plane.

**Figure 3:**
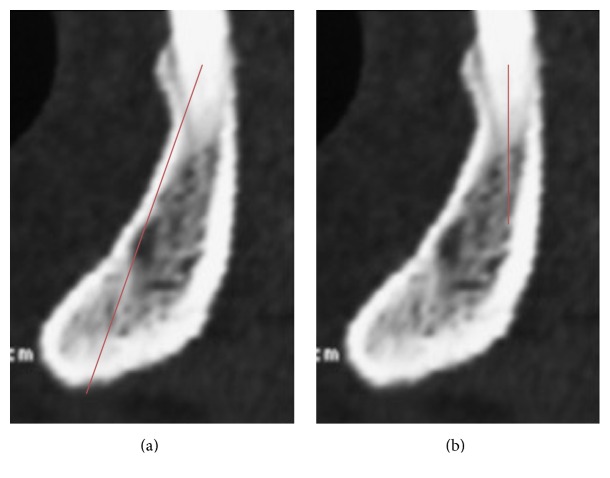
(a) The distance between the most coronal and medial point of the ridge and the lowest point along the axis of the crest; (b) the available distance to place the implant.

In CTs without teeth, the section between the apophysis genii was considered as the median one. The other measurements were chosen after measuring the mean distance of the areas considered in the CT with teeth with respect to the central section. For each region, the following measurements were observed: the axial height, vertical height, and angulation (Figures 2(a), 2(b), and 2(c)). In the interforaminal region, the axial height and the available distance to place the implant were compared (Figures 3(a) and 3(b)).

**Figure 4:**
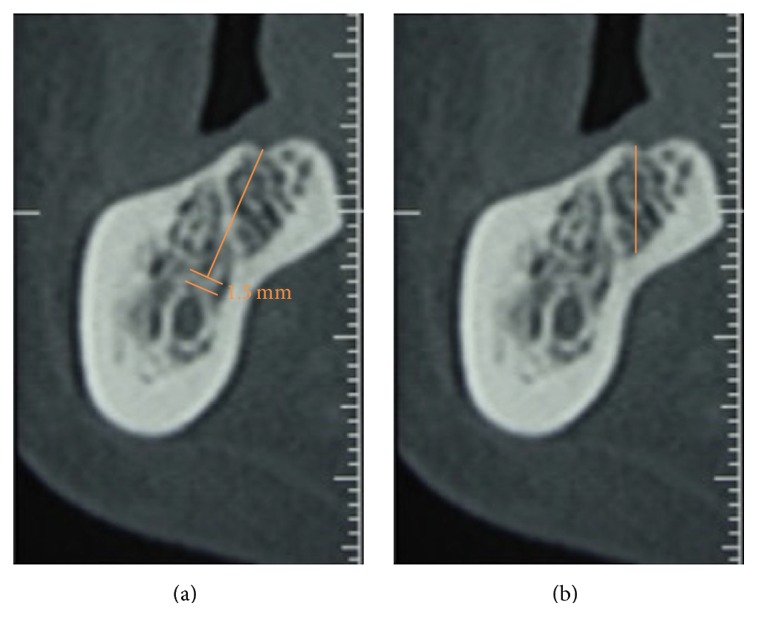
(a) The distance from the most coronal and medial point of the ridge to the inferior alveolar nerve; (b) the available distance for implant positioning.

In the posterior region of the mandible, considering a safety distance of 1.5 mm [19, 20], the distance from the most coronal medial point of the ridge to the inferior alveolar nerve was compared with the available distance for implant positioning (Figures 4(a) and 4(b)).

The available distance in both regions was chosen by one single expert implantologist as the maximum distance to place the implant according to an axis of insertion that realistically is close to the ideal one. Finally, given that the measurements for each sample were similar to each other, they were grouped into a single sample. Except for the symphysis region, the right and left sites of each region were combined into one single sample.

### 2.3. Statistical Analysis

The statistical analysis was performed using the statistical software package IBM SPSS Statistics for Windows, version 21.0 (Released 2012, IBM Corp., Armonk, NY, USA).

## 3. Results

One hundred and eighty-seven mandibular CTs (92 males and 95 females; mean age: 46.78 ± 14.36; age range: 18–70) were evaluated. The mandible presents a constant degree of angulation in the buccolingual direction and from the bottom upwards. The lowest degree of mandibular angulation was between the canine and first premolar in both groups (in the presence of teeth: mean 7.43° ± 4.77°; in the absence of teeth: mean 10.96 ± 4.99°). Therefore, the highest value was at the distal point of the second molar in both groups (in the presence of teeth: mean 16.06° ± 5.99°; in the absence of teeth: mean 16.37° ± 7.47°).

The mean angulation of the jaw in the samples examined in the absence of teeth is greater than in the presence of teeth in all the considered regions but the difference is statistically significant only in the region between the canine and first premolar and between the second premolar and the first molar (Table 1). There is no statistically significant difference in the angulation between women and men.

The difference between the axial height and the available distance to place the implant, considering the symphysis, is similar in both groups (Table 2). In the canine-first premolar region, more differences were observed between the values: in the presence of teeth: mean 3.67 mm; in the absence of teeth: mean 5.30 mm (Table 3).

Studying the posterior region, in the presence of teeth, the difference between the nerve distance and the available distance is greater in the distal site of the second molar (mean 2.46 mm) than in the second premolar-first molar region (mean 2.11 mm) (Table 4(a)). There is no difference between the two regions when the teeth are absent (Table 4(b)).

## 4. Discussion

Influencing factors, such as the buccolingual, apicocoronal, and mesiodistal 3D positions as well as the implant angulation, dictate a correct implant placement [21]. The ideal implant position must be predetermined according to the future prosthesis, occlusal plane, and esthetic parameters. Mandibular teeth, in the natural dentition, have a slight lingual inclination in relation to both the mandibular base [22] and the opposite maxillary arch dentition, and therefore implants should be placed considering both inclinations [23].

The results from our study showed that the mandibular angulation was 7.43° ± 4.77° in the presence of teeth and 10.96° ± 4.99° when the teeth were absent between the canine and first premolar. The same trend was also noted in the second premolar-first molar area. As presented, we noticed a major change of angulation, in the sense that the axial height and available distance were different from each other. This sets a restriction on the implant positioning according to an ideal prosthetic axis, limiting the use of angled abutments.

Angled abutments result in an increased stress on the implants and adjacent teeth [24]. When the abutment angulation increased, the stress and strain increased [25]. Although Sethi et al. reported that the magnitude of the angles did not significantly influence the overall implant survival rate [26], the stresses on the bone might go beyond physiological limits when the abutments are angled [27].

The anatomy of the mandible is different in the presence and absence of teeth and changes, due to the bone remodeling after tooth loss leading to a variation of the bone height, width, and angle, may complicate the surgical act of the implant placement [28]. Initially, the greatest amount of bone loss is in the horizontal dimension and occurs mainly on the buccal plate of the ridge. Our results, which consist in increased mandibular angulation in each considered region, are confirmed by the literature, which shows more buccal resorption and lingual relocation of the ridge [29]. These changes seem to be more evident in the middle regions of the mandibular body.

Although the area of second molar is not affected by excessive changes, overangulation, as mentioned before, may increase the difficulty in achieving an optimal implant placement. In the symphysis region, not having excessive angulation or an excessive modification of height and angulation after tooth loss, the axial height and available distance are similar in both groups, rendering this region a safety area, if a careful evaluation of the anatomical characteristics of the lingual plate has already been performed.

Some authors have even suggested that a CT scan should be performed routinely before implant placement in the interforaminal region [30].

In terms of image quality, reproducibility, and validity, the CBCT produced superior images to the helical CT, with approximately 400-fold less radiation exposure in the dental radiology field [31–33].

Another advantage is accuracy [1, 33] because the CBCT volumetric data is isotropic. This makes it possible to reorient the images to fit the patient's anatomical features and to perform real-time measurements [33, 34].

CBCT units provide choices in terms of field of view (FOV), which allows irradiation of particular areas of interest to dentists, while limiting the irradiation of other tissues. This function contributes to an excellent resolution and a minimal radiation risk for the patients [33, 35, 36].

One major disadvantage is that it can only demonstrate a limited contrast resolution [33, 37, 38]. If the objective of the examination is hard tissue only, using a CBCT would not be a problem; however, it is not sufficient for soft tissue evaluation [33, 39].

According to our measurements, it was possible to propose a classification of mandible angles based on the potential risk of lingual cortical plate perforation (Table 5):

- Low mandibular inclination (LMI): angle < 10°.
- Medium mandibular inclination (MMI): angle between 10° and 17°.
- High mandibular inclination (HMI): angle > 17°.

**Figure 5:**
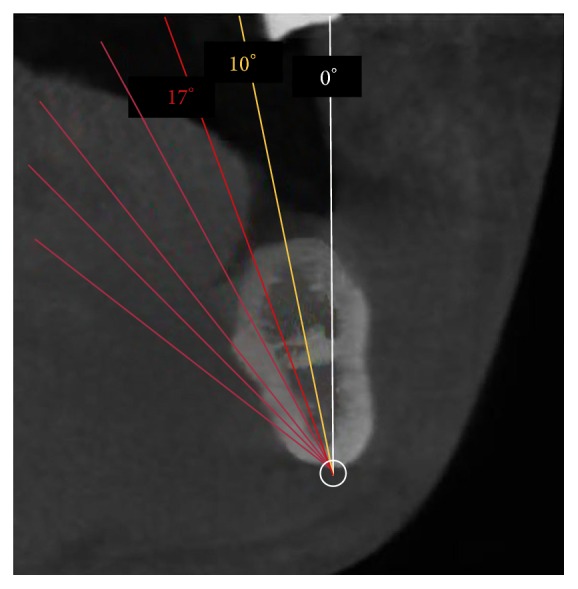
Different ranges of angulation.

One of the most important clinical applications of our study concerns postextraction implants; in these cases, clinicians usually believe that the transversal dimension is sufficient, given the postextractive nature of the implant. Moreover, clinicians assume that the vertical dimension can be calculated by using a panoramic radiograph, with a template to overcome the related distortion, adding at least 2 mm beyond the alveolar socket in order to achieve an optimal primary stability [40]. However, in the presence of high alveolar crest angulation, there is an increased risk of lingual plate perforation (Figure 5) which may trigger clinical complications.

Even today, many dentists in immediate postextractive implantology believe that the vertical dimension is present within the two-dimensionality of the OPT. However, due to the variability of the mandibular anatomy, CBCT, nowadays, is recommended for safer presurgery planning.

## 5. Conclusions

To prevent unintentional hemorrhages, in cases involving immediate placement, practitioners are recommended not to use the extraction socket as a guide for angulation. Alveolar bone resorption after tooth loss can be considered as a risk factor for lingual cortical perforation during the insertion of an implant. Presurgical implant planning is not possible without a proper study of a 3D radiographic examination that allows the clinician to study the mandibular anatomy and identify the risk areas.

## Figures and Tables

**Table 1:** Angulation means revealing statistically significant difference between the canine-first premolar region and second premolar-first molar region relating to the presence or absence of teeth.

	Presence of teeth	Absence of teeth	*P* value
Symphysis	(*n* = 111) 9.26 ± 6.63	(*n* = 76) 11.07 ± 8.84	0.374
Canine-first premolar	(*n* = 270) 7.43 ± 4.77	(*n* = 104) 10.96 ± 4.99	0.000632
Second premolar-first molar	(*n* = 252) 9.38 ± 5.38	(*n* = 122) 12.49 ± 5.94	0.000617
Distal site of second molar	(*n* = 178) 16.06 ± 5.99	(*n* = 196) 16.37 ± 7.47	0.768

**Table 2:** Difference between the axial height and the available distance at the level of symphysis revealing similarity in both groups (presence or absence of teeth).

	Presence of teeth	Absence of teeth
Axial height	28.23 ± 5.32	26.84 ± 4.67
Available distance	24.54 ± 4.73	22.90 ± 3.78

*Difference*	*3.69*	*3.94*

**Table 3:** Difference between the axial height and the available distance at the level of the canine-first premolar.

	Presence of teeth	Absence of teeth
Axial height	27.12 ± 3.62	24.22 ± 4.13
Available distance	23.45 ± 4.50	19.66 ± 3.93

*Difference*	*3.67*	*5.30*

**(a):** 

	Distance from the nerve (mm)	Available distance (mm)	Difference
Regions 5-6 (252 samples)	16.94 ± 4.17	14.83 ± 3.55	2.11
Region 7 (178 samples)	15.10 ± 3.93	12.64 ± 4.21	2.46

**(b):** 

	Distance from the nerve (mm)	Available distance (mm)	Difference
Regions 5-6 (122 samples)	14.32 ± 3.45	12.16 ± 3.56	2.16
Region 7 (196 samples)	12.6 ± 4.11	10.44 ± 3.72	2.16

**Table 5:** The proposed classification of mandibular angulation based upon the potential risk of lingual plate perforation.

	Angle	Risk assessment
LMI	<10°	Low risk
MMI	10° < angle < 17°	Medium risk
*HMI*	*>17*°	*High risk*

## Additional Points

The aim of this study was to evaluate the variability of the mandibular anatomy in the presence and absence of teeth and to consider how it could influence implant planning. The mandibular anatomy variability, due to the jaw angulation, presents challenges to the surgeon in the placing of the implant safely. To avoid any potential intra/postoperative complications, high precision radiography is recommended for better presurgery planning.
